# Pain resilience dimensions and regional gray matter volume as risk factors for poor outcomes of chronic pain: a prospective cohort study

**DOI:** 10.1017/S0033291724001703

**Published:** 2024-10

**Authors:** Beibei You, Hongwei Wen, Todd Jackson

**Affiliations:** 1 School of Nursing, Guizhou Medical University, Guiyang, 561113, China; 2 Key Laboratory of Cognition and Personality (Ministry of Education), Faculty of Psychology, Southwest University, Chongqing, 400715, China; 3 Department of Psychology, University of Macau, Taipa, 999078, Macau, SAR, China

**Keywords:** chronic pain, gray matter volume, longitudinal, mediation, pain resilience

## Abstract

**Background:**

Pain resilience and regional gray matter volume (rGMV) are established correlates of adaptation to chronic pain within cross-sectional studies. Extending such work, this prospective cohort study tested the status of baseline pain resilience dimension scores and rGMV as risk factors for subsequent exacerbations in chronic pain disability and intensity.

**Methods:**

142 adults with chronic musculoskeletal pain completed an initial assessment comprising a structural magnetic resonance imaging scan and self-report measures of cognitive/affective positivity and behavioral perseverance pain resilience dimensions, disability, pain intensity, and demographics. Disability and pain intensity were outcomes re-assessed at a 6-month follow-up. The impact of pain resilience dimension scores and identified rGMV sites on follow-up outcomes was examined after controlling for other baseline correlates of outcomes. Mediating effects of identified rGMV sites on pain resilience dimension-follow-up outcome relations were also evaluated.

**Results:**

Aside from the significant multivariate effect of lower behavioral perseverance and cognitive/affective positivity scores, augmented left precuneus, temporal pole, superior temporal gyrus (STG), and precentral gyrus rGMV combined to predict higher follow-up disability levels, independent of covariates. Higher left fusiform gyrus rGMV levels predicted follow-up exacerbations in pain intensity, but pain resilience dimension scores did not. Finally, left precuneus and left temporal pole STG rGMV partially mediated cognitive/affective positivity-follow-up disability relations.

**Conclusions:**

Findings underscore deficits in pain resilience and increased rGMV as potential risk factors for poorer subsequent outcomes of chronic musculoskeletal pain and provide foundations for further prospective extensions as well as targeted intervention research.

Chronic pain is widespread and potentially debilitating (Cohen, Vase, & Hooten, 2021; Hartvigsen et al., 2018), yet there is significant variability in how well people adapt to ongoing pain. Consequently, researchers have shown considerable interest in understanding factors that affect adjustment to pain. Individual differences in resilience – the capacity to resist or cope with challenges so that healthy physical and psychological functioning is maintained (e.g. Pascual-Leone & Bartres-Faz, 2021) – are one key influence on responses to adversity. Biological, social, and psychological formulations of resilience have proliferated over time. For example, some biological conceptualizations have underscored the brain's remarkable capacity to display structural and functional plasticity in response to stressful events via neuronal replacement, dendritic remodeling, and synapse turnover. Particular social-contextual perspectives emphasize resilience as a competency through which social interactions between people bolster personal and community resources to manage adversity (e.g. Sturgeon & Zautra, 2010). Popular psychological accounts have highlighted resilience as the capacity to successfully cope with internal and external stressors (e.g. Connor & Davidson, 2003).

Regarding pain-specific formulations, Slepian, Ankawi, Himawan, and France (2016) conceived of pain resilience as a trait-like protective psychological influence that mitigates negative effects of pain. To these authors, pain resilience comprises dimensions reflecting capacities to (i) maintain a positive outlook in regulating one's thoughts and emotions (cognitive/ affective positivity) and (ii) sustain engagement in daily activities (behavioral perseverance) despite ongoing pain. Higher scores on these dimensions correlate with lower intensity ratings and better tolerance for certain types of noxious laboratory stimuli (Ankawi, Slepian, Himawan, & France, 2020; Slepian et al., 2016; Slepian & France, 2017; You, Wen, & Jackson, 2024). Construct validity support for pain resilience cognitive/affective positivity and behavioral perseverance dimensions is also evidenced by positive correlations with conceptually related measures of general resilience, positive affect, pain acceptance, pain self-efficacy and quality of life as well as negative correlations with pain-related disability/impairment, pain intensity, pain catastrophizing, and emotional distress (Ankawi, Slepian, Himawan, & France, 2017; You & Jackson, 2021a). In longitudinal chronic pain research, total initial pain resilience scores had negative bivariate relations with physical dysfunction and depression, but not pain intensity (*r* = −0.04, *p* < ns) three months later (Slepian, Ankawi, & France, 2020).

Notwithstanding the need for prospective research on links between these pain resilience dimensions and later chronic pain outcomes, little is known about brain structure correlates of pain resilience or follow-up pain outcomes. Regional gray matter volume (rGMV) abnormalities are viewed as a potential risk factor for (e.g. Farrell et al., 2021) and consequence of (e.g. Rodriguez-Raecke, Niemeier, Ihle, Ruether, & May, 2009) chronic pain. Reviewers have concluded that chronic pain samples (*v.* pain-free controls) display significantly less rGMV in so-called ‘pain matrix’ structures, the default-mode network (DMN) and salience network as well as comparatively more rGMV in the right striatum, right hippocampus, right parahippocampal gyrus, and amygdala (e.g. Brandl et al., 2022; Ma et al., 2022; Smallwood et al., 2013; Wang et al., 2022). Furthermore, chronic pain intensity has negative correlations with anterior cingulate gyrus, insula, superior frontal gyrus, temporal gyrus, and hippocampus rGMV (e.g. Brandl et al., 2022; Coppieters et al., 2016; Wang et al., 2022) in addition to positive correlations with thalamus and post-central gyrus rGMV (Wang et al., 2022). While these reviews underscore structural abnormalities linked to the presence and intensity of chronic pain, they do not directly elucidate rGMV relations with pain resilience or future pain disability.

Nonetheless, several cross-sectional pain studies have explored rGMV-resilience associations. In early human laboratory pain research, Erpelding and Davis (2013) identified less *v.* more resilient subgroups from task performance interrupted by painful stimulation; less resilient cohorts had comparatively more rGMV in areas implicated in pain perception and salience (i.e. anterior insula, anterior mid-cingulate cortex, supplementary motor area, orbitofrontal cortex). Subsequently, Li and Jackson (2020) found lower resilience levels, based on Connor-Davidson Resilience Scale (CDRS; 2003) scores and cold pressor test performance, correlated with more rGMV in the left post-central gyrus, a structure implicated in the resilience-coping network (Sinha, Lacadie, Constable, & Seo, 2016), and the right inferior temporal gyrus, another pain processing region.

Within chronic pain samples, You and Jackson (2021b) created less *v.* more resilient groups based on total Pain Resilience Scale-Chinese and CDRS scores. Less resilient cohorts displayed comparatively smaller rGMV in self-referential processing, emotion regulation and executive control areas (bilateral precuneus, left superior and inferior parietal lobules, orbital right middle frontal gyrus, medial right superior frontal gyrus, bilateral median cingulate gyrus). Finally, within a mixed chronic pain sample, Hector et al. (2023) found higher general resilience questionnaire scores correlated with more rGMV in the rostral and subgenual anterior cingulate cortex (rACC, sgACC) and left dorsolateral prefrontal cortex. Right rACC and left sgACC GMV also partially mediated resilience-pain intensity relations.

Together, cross-sectional evidence suggests that resilience to pain is associated with rGMV in diverse brain regions involved in pain processing, emotion regulation, and executive control. However, it not clear whether or how rGMV is linked to cognitive/affective positivity and behavioral perseverance dimensions of pain resilience or later adaptation to chronic pain. To address these gaps, the initial aim of this prospective cohort study was to evaluate the status of baseline pain resilience dimension scores and rGMV as univariate and multivariate risk factors for poor subsequent chronic pain outcomes. Based on the preceding overview, we hypothesized that (1) lower cognitive/affective positivity and behavioral perseverance levels as well as (2) rGMV in structures involved in pain processing, executive control and emotion regulation would increase risk for exacerbations in disability and pain intensity at a six-month follow-up.

Second, extending cross-sectional tests of resilience-rGMV-chronic pain intensity mediation models (Hector et al., 2023), we assessed mediating effects of identified rGMV sites on prospective pain resilience dimension-rGMV-follow-up pain outcome relations. Specifically, we tested the exploratory hypothesis that negative associations of cognitive/affective positivity and behavioral perseverance dimension scores with follow-up disability and pain intensity would be partially mediated by identified rGMV correlates of these outcomes.

## Method

### Participants

The sample comprised community dwellers (92 women, 50 men) with chronic musculoskeletal pain. On average, participants were middle-aged and had a mean pain duration of slightly over seven years. Primary pain sites included back (33%), extremity (29%), neck or cervical spine (22%) and shoulder (16%), though over 80% acknowledged more than one pain site. Sample characteristics are summarized in online Supplementary Table S1.

### Procedure

The Human Research Ethics Committee of Southwest University, Chongqing, approved this research. Study procedures met ethical standards of the 2008 Helsinki Declaration. Selection criteria included an age of 18 years or older, chronic non-cancer musculoskeletal pain every day or most days for at least three months, no major psychiatric diagnoses that could hinder comprehension (e.g. psychotic disorders, bipolar disorder, dementia), non-use of opioid medication for pain relief, and literacy with written Chinese. Those with contraindications for magnetic resonance imaging (MRI) (i.e. pacemakers, metal implants, severe claustrophobia) were also excluded.

The study was part of a larger project on chronic pain in China. After receiving permission from various community settings, volunteers were solicited through print advertisements and referrals from setting staff. Among those who met selection criteria and expressed continued interest in participation, baseline assessments were scheduled. Upon arrival, participants completed an informed consent and reconfirmed their eligibility. Next, structural MRI scans were performed followed by completion of a brief questionnaire battery described below. At the six-month follow-up, participants were re-contacted and completed a brief structured phone interview to re-assess self-reported disability and pain intensity.

Of 162 participants who completed the baseline assessment, those whose primary pain site was the head or face (*n* = 10) or whose scan resulted in sub-optimal image quality (*n* = 9) were excluded. One other participant who lost contact with the study at follow-up was also excluded. The final sample comprised 142 respondents.

### Self-report measures

#### Pain resilience scale-Chinese (PRS-C; You and Jackson, 2021a)

The 10-item PRS-C assesses resilience to ongoing pain and includes 7-item cognitive/affective positivity and 3-item behavioral perseverance subscales. PRS-C items were rated from *0* *=* *not at all* to *4* *=* *all the time* and summed to calculate total subscale scores. The PRS-C largely replicated the original two-factor structure (Slepian et al., 2016) and has satisfactory psychometrics in Chinese chronic pain samples (You & Jackson, 2021a). In this study, baseline alphas were *α* = 0.84 and *α* = 0.85 for cognitive/affective positivity and behavioral perseverance, respectively.

#### Graded chronic pain scale-Chinese (GCPS-C; Von Korff, Ormel, Keefe, & Dworkin, 1992; Wong & Fielding, 2011)

The GCPS-C includes (i) a 3-item disability scale that examines interference with daily, social, and work activities during the past 3 months and (ii) a 3-item pain intensity subscale that queries average, current, and worst pain experienced during the same interval. Items were rated from *0* (*no disability/pain at all*) to *10* (*disability/pain as bad as it could be*). The GCPS-C factor structure and validity are supported within Chinese chronic pain samples (e.g. Wong and Fielding, 2011). In this study, alphas were acceptable for disability (Baseline: *α* = 0.90, Follow-up: *α* = 0.89) and pain intensity (Baseline: *α* = 0.82, Follow-up: *α* = 0.82) subscales.

#### Background characteristics

Demographics measures included gender, age, relationship status, ethnicity, education, number of dependents, height/weight for body mass index (BMI) calculation, and total intracranial volume (TIV). Pain factors assessed included pain duration (months), primary pain site, presence of other pain sites, and non-opioid prescription analgesic use.

### Image acquisition

Structural MRI scans were performed with a 3 T Trio scanner from Siemens Medical Systems (Erlangen, Germany). A magnetization prepared rapid acquisition gradient-echo sequence captured high-resolution BL-weighted structural images with the following parameters: repetition time/echo time = 2530 ms/3.39 ms, inversion time of 1100 ms, flip angle of 7°, field of view = 256 × 256 mm^2^, with 128 slices, slice thickness = 1.33 mm, and voxel size = 1 × 1 × 1.33 mm^3^. Prior to scanning, participants were instructed to lie still, relax, and think of nothing in particular. Soft foam pads and earplugs were provided to mitigate head movements and scanner noise.

### Image preprocessing

Structural images were preprocessed using Statistical Parametric Mapping (SPM 12; http://www.fil.ion.ucl.ac.uk/spm) software. Each image was evaluated for potential artifacts or pronounced anatomical anomalies, reoriented, and centered on the anterior commissure to optimize registration. We employed unified segmentation with light regularization and a priori tissue probability maps (TPMs) to plot gray matter (GM), white matter (WM), and cerebrospinal fluid. Advanced diffeomorphic anatomical registration based on exponentiated lie algebra (DARTEL) in SPM (Ashburner, 2007) was then used for segmentation, registration, normalization, and modulation. A study-specific template was derived from resliced GM and WM images using DARTEL to enhance alignment. Image voxel intensities were modulated using Jacobian determinants to preserve rGMV differences. Registered images were transformed to Montreal Neurological Institute (MNI) coordinate space and smoothed with an 8-mm full-width-at-half-maximum (FWHM) Gaussian kernel to normalize data and minimize inter-participant anatomical variability.

### Design and data analysis

#### Preliminary analyses

Spearmen correlation analyses were performed to identify baseline demographics and pain factors that had significant (*p* < 0.05) bivariate associations with follow-up disability and pain intensity levels. Follow-up elevations in disability were related to female gender (*r* = −0.17, *p* < 0.05), baseline elevations in disability (*r* = 0.47, *p* < 0.001) and pain intensity (*r* = 0.43, *p* < 0.001), and use of prescription analgesics (*r* = 0.16, *p* = 0.05). These correlates were controlled as covariates in all subsequent analyses involving follow-up disability. Higher follow-up pain intensity levels were correlated with female gender (*r* = −0.19, *p* < 0.05), baseline elevations in pain intensity (*r* = 0.52, *p* < 0.001) and disability (*r* = 0.41, *p* < 0.001), and having multiple pain sites (*r* = 0.19, *p* < 0.05) which served as covariates within subsequent analyses involving follow-up pain intensity.

#### Main analyses

Partial correlation analyses examined the status of lower pain resilience cognitive/affective positivity and behavioral perseverance dimension scores as univariate risk factors for follow-up exacerbations in disability and pain intensity, after statistically controlling for relevant baseline covariates identified in preliminary analyses. Similarly, to identify univariate rGMV risk factors for follow-up increases in disability and pain intensity, partial correlation analyses were run in SPM. All significant rGMV clusters were identified by applying a Gaussian random field (GRF) correction (voxel-level *p* < 0.001, cluster-level *p* < 0.05) (Fu et al., 2019; Kong et al., 2018). Significant sites were designated as regions of interest (ROIs). Average ROI values were subsequently extracted via the Response Exploration (REX) tool (https://www.nitrc.org/projects/rex/). In these analyses, significant rGMV-follow-up disability and rGMV-follow-up pain intensity partial correlations were examined after controlling for baseline covariates noted above, age and TIV.

Hierarchical multiple regression analyses tested the hypothesis that lower pain resilience cognitive/affective positivity and behavioral perseverance dimension scores as well as identified rGMV sites would be univariate and multivariate risk factors for follow-up elevations in chronic pain (i) disability and (ii) pain intensity. In each regression model, one six-month follow-up outcome (either disability or pain intensity) was the dependent measure. Block 1 of each model comprised relevant baseline covariates of the follow-up outcome identified in preliminary analyses. After controlling for Block 1 covariates, we evaluated univariate and multivariate effects of pain resilience dimension scores (Block 2) and significant rGMV values (Block 3) as predictors.

Finally, mediation analyses tested the exploratory hypothesis that identified rGMV sites would partially mediate statistically significant pain resilience dimension-follow-up outcome associations identified within partial correlation analyses. In each analysis, a Simple Mediation Model (model 4) of the Hayes Process macro for SPSS (Hayes, 2012) was used. Furthermore, bootstrapping with 5000 resamples was used to estimate indirect effects against zero by establishing confidence intervals (CIs). Effects were significant at *p* < 0.05 following Hector et al. (2023). Within each mediation model, baseline covariates of each follow-up outcome specified above were controlled so that unique relations between the relevant pain resilience dimension, rGMV site, and follow-up outcome could be elucidated.

## Results

### Partial correlations between pain resilience dimensions and follow-up outcomes

Table 1 presents partial correlations of associations between each pain resilience dimension and follow-up pain outcome, after controlling for relevant covariates. Cognitive/affective positivity and behavioral perseverance were univariate risk factors for follow-up disability levels based on significant negative partial correlations. In contrast, resilience dimension-follow-up pain intensity partial correlations were not significant.

**Table 1.** Partial correlations of baseline (BL) pain resilience dimensions, and identified gray matter volume regions of interest (rGMV) with disability and pain intensity at the six-month follow-up (6mo) (*N* = 142)

Measure	1	2	3	4	5	6	7	8
1. 6mo disability	–							
2. 6mo pain intensity	0.68***	–						
3. BL cognitive-affective positivity dimension of pain resilience	−0.24**	−0.08	–					
4. BL behavior perseverance dimension of pain resilience	−0.30***	−0.15	0.54***	–				
5. BL left precuneus rGMV	0.25**	0.12	−0.22*	0.04	–			
6. BL left pre-central gyrus rGMV	0.26**	0.14	−0.14	0.05	0.60***	–		
7. BL left temporal pole: superior temporal gyrus rGMV	0.22**	0.01	−0.19*	−0.03	0.61***	0.65***	–	
8. BL left fusiform gyrus rGMV	0.13	0.25**	−0.06	0.06	0.63***	0.64***	0.67***	–

*Note*. *p < 0.05, **p < 0.01, ***p < 0.001. In partial correlation analyses involving 6mo disability, covariates were BL measures of pain disability, pain intensity, gender, and prescription analgesics use. In partial correlation analyses involving 6mo pain intensity, covariates were BL measures of pain intensity, disability, gender, and presence of other pain sites.

### Partial correlations of identified rGMV sites with follow-up outcomes and pain resilience dimensions

Location and composition details for significant rGMV correlates of follow-up pain outcomes are presented in Table 2 and online Supplementary Fig. S1. Left precuneus, left temporal pole: superior temporal gyrus (STG), and pre-central gyrus rGMV and had significant positive partial correlations with follow-up disability, supporting their status as univariate risk factors for this outcome (Table 1). Left precuneus and left STG rGMV also had significant negative partial correlations with cognitive/affective positivity scores (Table 1) and were also tested in cognitive/affective positivity-rGMV-follow-up disability mediation models.

**Table 2.** Baseline regional gray matter volume (rGMV) correlates of disability and pain intensity at six-month follow-up (*N* = 142)

Follow-up outcome	Gray matter volume in identified region	Brodmann area	Montreal neurological institute space			Cluster size	Voxel level
Disability	Left precuneus	7	−8	−54	42	224	0.30
	Left pre-central gyrus	6	−32	−8	59	148	0.33
	Left temporal pole: superior temporal gyrus	38	−41	9	−23	133	0.30
Pain intensity	Left fusiform gyrus	19	−41	−78	−20	117	0.31

*Note*. All voxel-level *ps* < 0.001, all cluster *ps* < 0.05. Results reflect partial correlations between rGMV in identified baseline brain regions and follow-up outcomes after controlling for baseline covariates of outcomes using a GRF corrections. In analyses of disability as the follow-up outcome, baseline measures of disability, pain intensity, gender, age, total intracranial volume, and prescription analgesics use were covariates. In analyses of pain intensity as the follow-up outcome, baseline measures of pain intensity, disability, gender, age, total intracranial volume, and presence of other pain sites were covariates.

Left fusiform gyrus (FG) rGMV was a univariate risk factor for increased follow-up pain intensity, based on its significant positive partial correlation with this outcome (Table 1). However, due to non-significant epartial correlations between FG rGMV and pain resilience dimensions (Table 1), mediation analyses for follow-up pain intensity were not warranted.

### Multivariate prediction model for follow-up disability

The hypothesis that lower pain resilience dimension scores as well as identified rGMV sites would be risk factors for higher follow-up disability levels was partially supported within hierarchical multiple regression analyses. Together, lower cognitive/affective positivity and behavioral perseverance scores (Block 2) explained significant unique variance in follow-up disability elevations (*R^2^Ch.* = 0.08, *p* < 0.01), albeit only behavioral perseverance had a significant univariate effect (See Table 3). Furthermore, more pronounced rGMV in the left pre-central gyrus, left precuneus and left temporal pole STG (Block 3) combined to predict significantly higher disability levels at follow-up (*R^2^Ch.* = 0.06, *p* < 0.01), though univariate rGMV effects were not significant (Table 3).

**Table 3.** The impact of baseline demographics, pain experiences, pain resilience, and regional gray matter volume (rGMV) on six-month follow-up disability levels (*N* = 142)

		Criterion measure	
		Follow-up disability	
Block	Baseline predictor	*β*(CI 95%) *t*	*t*
1	Gender	−0.07 (−0.22 to 0.08)	−0.90
	Prescription analgesics use	0.05 (−0.10 to 0.20)	0.62
	Pain intensity	0.21 (0.01–0.40)	2.04*
	Disability	0.34 (0.14–0.53)	3.41**
	Multivariate effect for Block 1: *R^2^* Change = 0.28, *F* Change (4, 137) = 13.16***		
2	Behavior perseverance	−0.24 (−0.41 to −0.07)	−2.75**
	Cognitive/affective positivity	−0.13 (−0.30 to 0.05)	−1.46
	Multivariate effect for Block 2: *R^2^* Change = 0.08, *F* Change (2, 135) = 8.03**		
3	Left pre-central rGMV	0.16 (−0.03 to 0.34)	1.65
	Left precuneus rGMV	0.12 (−0.07 to 0.31)	1.26
	Left temporal pole: superior temporal gyrus rGMV	0.01 (−0.19 to 0.21)	0.11
	Multivariate effect for Block 3: *R^2^* Change = 0.06, *F* Change (3, 132) = 4.18**		
Overall prediction model: *R^2^* *=* *0*.41, *F*_(9, 132)_ = 10.21***			

*Note*. *p < 0.05, **p < 0.01, ***p < 0.001, *β*, standardized beta coefficient; *CI*, confidence interval.

### Multivariate prediction model for follow-up pain intensity

The hypothesis that lower pain resilience dimension scores and identified rGMV sites would predict higher follow-up pain intensity levels received limited support (Table 4). Higher baseline pain intensity levels were the sole significant univariate predictor of follow-up pain intensity elevations in Block 1 of the model. Conversely, pain resilience dimension scores (Block 2) failed to explain significant additional variance in this outcome, either individually or in tandem. However, after controlling for all other predictors, more pronounced left FG rGMV (Block 3) was a significant risk factor for higher follow-up pain intensity relevels (*R^2^Ch.* = 0.05, *p* < 0.01) (see Table 4).

**Table 4.** The impact of baseline demographics, pain experiences, pain resilience and regional gray matter volume (rGMV) on six-month follow-up pain intensity levels (*N* = 142)

Measure		Criterion measure	
		Follow-up pain intensity	
Block	Baseline predictor	*β* (CI 95%)	*t*
1	Gender	−0.08 (−0.23 to 0.08)	−0.99
	Presence of other pain sites	0.06 (−0.09 to 0.22)	0.78
	Pain intensity	0.40 (0.20–0.60)	3.95**
	Disability	0.13 (−0.07 to 0.32)	1.28
	Multivariate effect for Block 1: *R^2^* Change *=* *0*.29, *F* Change (4, 137) *=* 13.75**		
2	Behavior perseverance	−0.14 (−0.31 to 0.04)	−1.51
	Cognitive/affective positivity	−0.01 (−0.19 to 0.17)	−0.15
	Multivariate effect for Block 2: *R^2^* Change = 0.02, *F* Change (2, 135) = 1.52		
3	Left fusiform rGMV	0.25 (0.09–0.40)	3.16*
	Multivariate effect for Block 3: *R^2^* Change = 0.05, *F* Change (1, 134) = 9.96*		
Overall prediction model: *R^2^* *=* *0*.35, *F*_(7, 134)_ = 10.33**			

*Note*. * *p* < 0.01, ** *p* < 0.001, *β*, standardized beta coefficient; *CI*, Confidence Interval.

### Mediating effects of rGMV on pain resilience dimension-follow-up outcome relations

**Figure 1.**
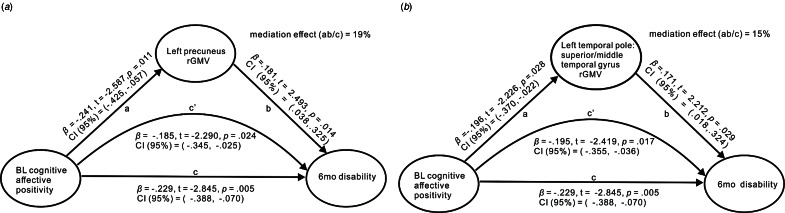
Mediation models of associations between baseline (BL) cognitive/affective positivity, BL regional gray matter volume (rGMV) in associated regions of interest, and 6-month follow-up (6mo) disability levels. *Note*. For Fig. 1a, the correlation between BL cognitive/affective positivity (predictor) and 6mo pain disability (outcome variable) was partially mediated by BL left precuneus rGMV (mediator) with gender, prescription analgesics use, BL pain severity, and BL pain disability as covariates. The indirect effect value was −0.044, *p* = 0.014, CI (95%) −0.113 to −0.004. For Fig. 1b, the correlation between BL cognitive/affective positivity (predictor) and 6mo pain disability (outcome variable) was significantly mediated by left temporal pole: superior/middle temporal gyrus rGMV (mediator) with gender, prescription analgesics use, BL pain severity, and BL pain disability as covariates. The indirect effect value was −0.034, *p* = 0.032, CI (95%) −0.097 to 0.001. ab, indirect effect; c’, direct effect; c, total effect. *β*, standardized beta coefficient; CI, confidence interval; BL, baseline; 6mo, Follow-up.

The exploratory hypothesis that rGMV would partially mediate significant pain resilience-follow-up outcome relations garnered support for two of four identified brain regions. Specifically, negative associations between cognitive/affective positivity and follow-up disability levels were partially mediated by more pronounced rGMV in the left precuneus (Fig. 1a) and left temporal pole: STG (Fig. 1b).

## Discussion

The main purpose of this research was to test the status of behavioral perseverance and cognitive/affective positivity dimensions of pain resilience and rGMV as risk factors for later changes in chronic pain-related disability and intensity using a prospective study design. Related analyses largely corroborated the initial hypothesis that lower pain resilience dimension scores as well as identified rGMV sites would predict subsequent exacerbations in chronic pain disability, independent of other significant baseline factors. However, support was more selective for follow-up pain intensity elevations as the outcome; neither univariate nor multivariate effects of pain resilience were significant but more pronounced rGMV in the left FG had a significant unique impact. The exploratory hypothesis that identified rGMV sites would mediate significant pain resilience-follow-up outcome relations garnered support, albeit mediation was specific to left precuneus and STG effects on cognitive/affective positivity-follow-up disability associations. Related implications of main findings are elaborated below.

### Pain resilience dimensions and rGMV as risk factors for disability exacerbations

Evidence for reduced cognitive/affective positivity and behavioral perseverance as risk factors for six-month follow-up disability elevations extends past research documenting a negative bivariate correlation between total pain resilience scores and physical dysfunction at a three-month follow-up (Slepian et al., 2020). This finding aligns with tenets of fear-avoidance (Vlaeyen, Crombez, & Linton, 2016) and cognitive appraisal (Jackson, Wang, & Fan, 2014a; Jackson, Wang, Wang, & Fan, 2014b) perspectives on chronic pain. Presumably, highly pain resilient cohorts more typically appraise pain as a challenge than a threat, maintain a positive outlook in managing their thoughts and emotions instead of ceding to fear or despair, and pursue, rather than avoid, valued goals, despite ongoing pain. For them, determined engagement in daily activities protects against fear, activity avoidance, physical disuse, and later disability (Jackson et al., 2014a, 2014b; Vlaeyen et al., 2016). While dysfunction and distress are essential foci of assessment and interventions to alleviate suffering, significant pain resilience-follow-up disability, associations observed here underscore the importance of also considering ‘protective’ psychological factors in assessment and treatment efforts to improve chronic pain outcomes.

Aside from pain resilience dimension scores, more pronounced left precuneus, left temporal pole: STG, and left precentral gyrus rGMV combined to explain significant exacerbations in disability at follow-up, independent of other baseline covariates and pain resilience dimension scores. Increased precuneus rGMV has been observed previously within distressed and/or disabled clinical samples (*v.* healthy controls) (e.g. Kong et al., 2015; Kroes, Rugg, Whalley, & Brewin, 2011; Tükel et al., 2015), including those with chronic pain (e.g. Chehadi et al., 2018; Luchtmann et al., 2014). The positive precuneus rGMV-disability partial correlation found here may reflect precuneus involvement in pain processing (Goffaux, Girard-Tremblay, Marchand, Daigle, & Whittingstall, 2014; Huber, Lui, & Porro, 2013), generation of pain expectations (Koyama, McHaffie, Laurienti, & Coghill, 2005), and/or pain catastrophizing/rumination (Seminowicz & Davis, 2006), based on its connectivity with the DMN (Utevsky, Smith, & Huettel, 2014). Moreover, pain chronicity is hypothesized to interfere with precuneus-DMN connectivity and the ability to shift attention away from pain (Alshelh et al., 2018; Cavanna & Trimble, 2006). Coupled with impaired attentional-switching capacities, Luchtmann et al. (2014) hypothesized that pronounced precuneus GMV reflects the structure's involvement in pain-related fear and avoidance of healthy activities that perpetuate chronic pain disability.

In tandem with other identified ROIs, increased rGMV in the left temporal pole STG also predicted higher subsequent cohort disability levels. While the STG has been neglected in pain imaging research because its status as an ROI is not obvious (Smallwood et al., 2013), augmented STG rGMV has been found in chronic pain patients *v.* controls (Chen et al., 2018a). Houde et al. (2020) posited that STG involvement in exaggerated painful memories contributes to maladaptive brain plasticity that fosters pain chronification. They found increased STG activity from painful electrical shock predicted exaggerated pain memories two months later while a ‘virtual lesion’ from transcranial magnetic stimulation over the STG during encoding of painful stimulation resulted in weaker subsequent memories of pain unpleasantness. Because more resilient adults direct less attention toward painful affective cues than neutral cues (Jackson, Yang, & Su, 2019; Ling, Yang, & Jackson, 2019; Zuo, Ling, & Jackson, 2023), they may be less prone to encoding and/or remembering pain stimuli. Supporting this contention, Zuo et al. (2023) found significantly poorer memory of painful injury images, but not happy images, among more (*v.* less) generally resilient adults. As such, pain attention and memory attenuations merit consideration in future hypothesis tests of STG rGMV-pain disability relations.

Finally, along with other rGMV correlates, increased pre-central gyrus rGMV predicted more subsequent chronic pain disability. Select studies have previously found more pre-central gyrus rGMV in chronic pain samples than pain-free controls (e.g. Riederer et al., 2017; Sundermann et al., 2019) The pre-central gyrus is involved in coordinating avoidance and defensive motor behaviors (e.g. freezing, withdrawal) of animals (Cooke & Graziano, 2004) and humans (Strawn et al., 2015) when potential threats loom. Furthermore, increased pre-central gyrus GMV has been implicated with impaired regulation of anticipatory threat stimuli (Liu et al., 2023). These data suggest avoidant responses to perceived threats of pain are one plausible functional basis for pre-central gyrus contributions to the multivariate rGMV-follow-up disability association we observed.

### Pain resilience dimensions and rGMV as risk factors for pain intensity exacerbations

The significant positive association between pain intensity ratings between baseline and follow-up aligns with other longitudinal evidence (e.g. Jackson et al., 2019) and underscores considerable stability in subjective judgments of chronic pain intensity over time. In contrast to cross-sectional support for associations between pain resilience dimensions and chronic pain intensity (e.g. Ankawi et al., 2017; You & Jackson, 2021a), baseline pain resilience dimension-follow-up pain intensity associations were not significant across univariate and multivariate analyses in line with previous longitudinal research (Slepian et al., 2020). Pain resilience taps capacities to maintain a positive outlook and persevere at tasks *despite* ongoing pain, not an ability to reduce, control or eliminate pain itself. Hence, elevated pain resilience may be more directly relevant to living well despite pain than to how much pain one experiences over time.

Increased left FG rGMV was the sole significant biomarker for follow-up exacerbations in pain intensity, extending related cross-sectional evidence based upon community dwellers (Ruscheweyh, Wersching, Kugel, Sundermann, & Teuber, 2018) and chronic pain patients (Niddam, Lee, Su, & Chan, 2017). Although the FG is widely implicated in visual processing, more pronounced FG rGMV has been documented in various chronic pain (*v.* pain-free control) conditions (e.g. Li, Zhang, & Kurata, 2018; Luchtmann et al., 2014; Riederer et al., 2017; Ung et al., 2014). FG involvement in emotion regulation is one plausible functional basis for its pronounced relationship with pain intensity. For example, increased FG activation corresponds to exaggerated reports of pain and/or emotion dysregulation in clinical pain samples (Stoeter et al., 2007; Su et al., 2015) while pronounced FG rGMV has been linked to over-identification with negative emotions (Guan et al., 2021) and poor use of emotions in problem-solving (Tan et al., 2014). Such emotion regulation deficits may foster perceptions of more intense pain.

### rGMV as a mediator of pain resilience dimension-follow-up disability relations

Mediation analyses indicated a negative cognitive/affective positivity-follow-up disability association was partially mediated by augmented left precuneus rGMV. The significant cognitive/affective positivity-precuneus rGMV relationship we observed dovetails with evidence linking a present-fatalistic time orientation, characterized by reduced optimism about the future, hopelessness and helplessness (Zimbardo & Boyd, 2015) to increased precuneus rGMV across two samples (Chen, Guo, & Feng, 2018b). These findings suggest precuneus involvement in maintaining optimistic expectations might affect how disabling chronic pain becomes over time. Increased rGMV in the left temporal pole STG also partially mediated the cognitive/affective positivity-follow-up disability association. Drawing from Houde et al.'s (2020) work elaborated above, STG involvement in attention to and encoding of painful *v.* non-painful information may help to explain why higher positivity levels predicted less subsequent disability. Mediating effects of precuneus and STG rGMV suggest that these structures reflect possible biological underpinnings for beneficial effects of cognitive/affective positivity in preventing exacerbations in pain-related disability, albeit their contributions are quite modest.

### Implications for research and practice

Aside from providing an impetus for future prospective studies, main findings have implications for assessment and treatment. Support for pain resilience dimension scores as risk factors for exacerbations in later disability encourages their inclusion within pain assessment protocols and suggests exposure-based pain management interventions (e.g. Vlaeyen & Crombez, 2020) to bolster behavioral perseverance and an optimistic orientation toward daily pursuits can reduce disability. The emergence of (i) left FG rGMV and (ii) left precuneus, STG, and precentral gyrus rGMV, respectively, as risk factors for exacerbations in pain intensity and disability at follow-up, suggests that these brain sites are viable targets in future treatment research. For example, investigating effects of brain stimulation and graded activity increases on these regions or related brain networks may elucidate clinically useful interventions for intractable chronic musculoskeletal pain.

### Limitations

Despite its implications, the main limitations of this study merit attention. First, causal effects of pain resilience and rGMV were not demonstrated because experimental designs featuring random assignment to specific conditions (e.g. resilience-building *v.* placebo control interventions) are needed to do so. Second, data were based on heterogeneous pain sites so findings may not apply to particular musculoskeletal disorders. Third, MRI data from several cohorts were lost due to poor image quality, in part, because we could not isolate head motion effects in image processing. Finally, to reduce response burdens and attrition risk, numerous correlates of pain and disability (e.g. trauma history, medication history, coping, social support) were not assessed. Extensions involving experimental study designs, homogeneous pain diagnoses and refined assessment approaches may remedy these limitations.

## Conclusion

In sum, this research established lower behavioral perseverance and cognitive/affective positivity pain resilience dimension scores as well as augmented left precuneus, STG and pre-central gyrus rGMV as multivariate risk factors for follow-up exacerbations in chronic pain disability. Increased left precuneus and STG rGMV also partially mediated cognitive/affective positivity-follow-up disability relations. Finally, higher left FG rGMV values emerged as a significant risk factor for later exacerbations in pain intensity, while the impact of pain resilience dimension scores was not significant. Findings provide empirical foundations for additional longitudinal studies and intervention research targeting pain resilience and localized rGMV as means of improving chronic pain outcomes.

## Supporting information

You et al. supplementary material You et al. supplementary material

## Supplementary material

The supplementary material for this article can be found at https://doi.org/10.1017/S0033291724001703
