# Supplementary material for: Pain resilience dimensions and regional gray matter volume as risk factors for poor outcomes of chronic pain: a prospective cohort study
Source: Psychol Med. 2024 Oct 23;54(13):3636–45. doi: 10.1017/S0033291724001703 (PMC11536115; doi:10.1017/S0033291724001703)
Supplement: You et al. supplementary material [file S0033291724001703sup001.docx]

Supplementary Table 1.

Sample characteristics at baseline and six-month follow-up outcomes (n = 142).

| Measure | *N* (%) / mean ± *SD* (range) |
| --- | --- |
| Gender (male) | 50 (35%) |
| Age (years) | 52.34 ± 17.04 (18 - 74) |
| Relationship Status |  |
| Single | 35 (25%) |
| In partner relationship | 107 (75%) |
| Ethnicity |  |
| Han majority | 141 (99%) |
| Ethnic minority | 1 (1%) |
| Education |  |
| Lower than high school completion | 44 (31%) |
| Higher than high school completion | 98 (69%) |
| Body mass index | 23.18 ± 3.02 (16.23 - 32.89) |
| Pain duration (months) | 86.49 ± 82.11 (5 - 387) |
| Presence of other pain sites (yes) | 117 (82%) |
| Total intracranial volume (TIV) (ml) | 1335.84 ± 127.96 (974.58 - 1701.67) |
| Prescription medication (yes) | 44 (31%) |
| Cognitive/affective positivity dimension of pain resilience | 20.02 ± 4.51 (4.00 - 28.00) |
| Behavioral perseverance dimension of pain resilience | 8.59 ± 2.45 (1.00 - 12.00) |
| Baseline disability level | 10.65 ± 5.17 (3.00 - 23.00) |
| Six-month follow-up disability level | 11.80 ± 5.22 (3.00 - 24.00) |
| Baseline pain intensity level | 13.94 ± 4.73 (5.00 - 25.00) |
| Six-month follow-up pain intensity level | 14.23 ± 4.46 (4.00 - 27.00) |

*Note.* Demographics, pain characteristics and pain resilience were assessed at BL.

| A  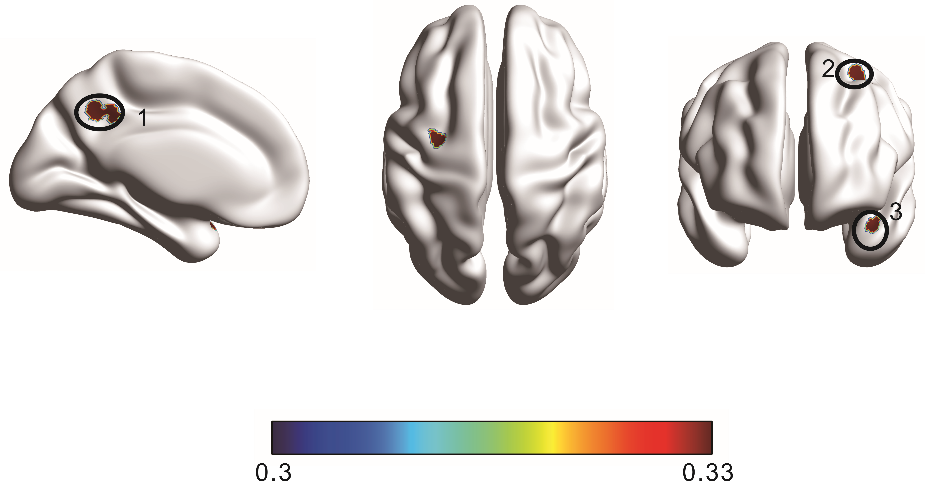 | B  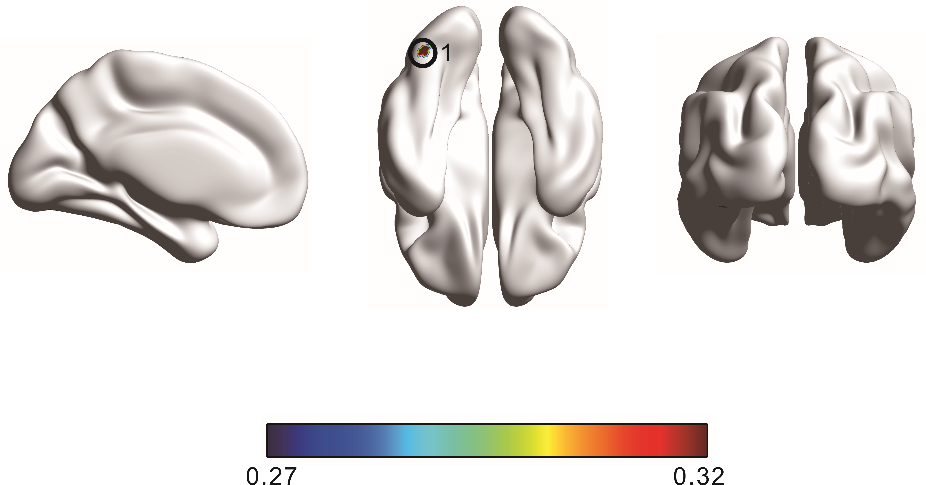 |
| --- | --- |

Supplementary Figure 1.

Brain regions in which regional gray matter volume (rGMV) values correlated with follow-up disability or pain intensity.

Notes. GRF correction, voxel-level *ps* < 0.001, cluster *ps* < 0.05. rGMV colors vary from blue (minimum correlation) to red (maximum correlation). Figure 1-A illustrates brain areas in which baseline rGMV values significantly correlated with pain disability at follow-up: 1 = left precuneus; 2 = left pre-central gyrus；3 = left temporal pole - superior/middle temporal gyrus. Figure 1-B illustrates brain areas in which baseline rGMV values significantly correlated with pain intensity at follow-up: 1 = left fusiform gyrus.
